# A pilot study to evaluate the role of circulation CD4^+^ CCR6^+^ CRTh2^+^ cell in predicting risk of asthma in wheezing children

**DOI:** 10.1186/s12887-021-02746-5

**Published:** 2021-06-05

**Authors:** Jingyang Li, Jinhong Wu, Haipei Liu, Li Hua, Quanhua Liu, Dingzhu Fang, Yi Chen, Ruoxu Ji, Jianhua Zhang, Wenwei Zhong

**Affiliations:** 1 Department of Pediatrics, Xinhua Hospital, Shanghai Jiao Tong University School of Medicine, 1665 Kongjiang Road, 200090 Shanghai, China; 2 Department of Pediatrics, Shanghai Children’s Medical Center, Shanghai Jiao Tong University School of Medicine, 1678 Dongfang Road, 200127 Shanghai, China

**Keywords:** Asthma, CD4^+^CCR6^+^CRTh2^+^ memory Th2 cell, Wheezing, Children

## Abstract

**Background:**

Wheezing is common in younger children and often related to viral infection. It is lack of reliable indicators for asthma prediction.

**Objective:**

To evaluate the relationship between circulation CD4^+^CCR6^+^CRTh2^+^ memory Th2 cells and asthma diagnosis in wheezing children.

**Methods:**

A prospective study was performed in children under 5 years old presented with wheezing or at last one episode of documented wheezing history. After inclusion, the level of serum allergen-specific serum IgE (sIgE) and circulating CD4^+^CCR6^+^CRTh2^+^cells were detected. The patients’ personal and family histories of allergic disease were acquired by questionnaire. The children were followed up over 2 years. Diagnosis of asthma was assessed at the end follow-up. The risk factors in predicting asthma diagnosis were evaluated.

**Results:**

A total of 43 children completed follow-up. Higher wheezing frequency were found in children with asthma diagnosis. The mean of circulating CD4^+^CCR6^+^CRTh2^+^cells in children diagnosed with or without asthma was 1.6 %±0.8 and 0.8 %±0.6 %, respectively, and was significantly higher in children diagnosed with asthma (*p* < 0.01). There was no significant difference between children with and without allergic diseases history or family allergic diseases in level of circulating CD4^+^CCR6^+^CRTh2^+^ cells. Logistic regression analysis indicated that circulating CD4^+^CCR6^+^CRTh2^+^ cells (EXP, 8.986; 95 % CI,1.886–42.816) and wheezing frequency(EXP, 0.127; 95 % CI, 0.023–0.703)were high risk factors for asthma.

**Conclusions:**

Our exploratory study shown that circulating CD4^+^CCR6^+^CRTh2^+^ memory Th2 cells increased in asthma diagnosed children and it was a high-risk factor for asthma. Detection of this type of cells could be helpful in predicting the risk of asthma in wheezing children.

## Background

Asthma is a common chronic inflammatory disease of the airway in children. The most typical symptom of asthma is recurrent wheezing. However, wheezing is common in younger children which is commonly related to acute respiratory tract infection. The Modified Asthma Predictive Index (mAPI) is a practical method commonly used in the clinic; however, its sensitivity and specificity are limited. Therefore, it is of great clinical value to explore biological indicators based on the immunology of bronchial asthma to guide the diagnosis and treatment of asthma more effectively.

Pathogenic memory type Th2 cells play an important role in allergic airway inflammation. CD4^+^ memory T cells can not only cause or aggravate allergic airway inflammation by inducing eosinophilic aggregation, mucus hypersecretion [1] and IL-17 secretion [2], but also by inducing airway hyperresponsiveness without eosinophil accumulation [3]. Conversely, inhibition of memory Th2 cells can alleviate allergic airway inflammation and improve the symptoms of asthma [4]. Those studies suggest that memory Th2 cells reflect previous allergic inflammation and can distinguish wheezing caused by non-allergic inflammation versus an immunological mechanism. CD4^+^ CCR6^+^ CRTh2^+^ cell is an important pathogenic memory type Th2 cells which is mainly resident in airway [5].

In this study, a cohort of wheezing children was established. The patient and family histories of allergic diseases were recorded in detail. In addition, the proportion of circulating CD4^+^CCR6^+^CRTh2^+^ memory Th2 cells were measured, and the wheezing children were followed up for 2 years to confirm whether they were diagnosed with asthma. Statistical methods were used to determine the correlation between the level of circulating CD4^+^CCR6^+^CRTh2^+^ memory Th2 cells and a diagnosis of asthma, thereby providing a theoretical basis for the early diagnosis of asthma in children.

## Results

### Subject characteristics and demographics

A total of 45 children were enrolled in this study. Among them, 43 cases (95.6 %) underwent blood tests and completed follow-up. 14 (32.6 %) of the included patients were girls. The median age of children at follow-up was 3.0 years and the age distribution was as follows: 21 cases were aged under 3-year-old (48.8 %) and 22 cases were 3–5 years old (51.2 %). 22 cases (51.2 %) presented lower respiratory tract infection (LRTI). 10 cases (23.3 %) of the children used systemic glucocorticoid because of wheezing. A total of 76.7 % of the subjects had other allergic diseases. 32 cases (74.4 %) positive for at least one food or inhalants sIgE. 23 cases (53.5 %) had a family history of allergic diseases. 36 cases (83.7 %) had more than two wheezing episodes at the end of follow-up (recurrent wheezing). 24 cases (55.8 %) accepted inhaled corticosteroids or montelukast therapy for more than 3 months. Characteristics of the patients are listed in Table 1. There was no significant difference between children with or without asthma diagnosis in age and sex (*p* > 0.05) (Table 2).

**Table 1:** Baseline characteristics of the study subjects

Characteristics	Study group (***n***=43)
Sex n (%)	
Male	29 (67.4)
Female	14 (32.6)
Age group (years, n (%))	
≤3 years old	21 (48.8)
3 -5 years old	22 (51.2)
Patients’ allergic disease n (%)	33 (76.7)
Family atopy history n (%)	23 (53.5)
Specific allergen n (%)	32 (74.4)
Recurrent wheezing n (%)	36 (83.7)
Controller Therapy^a^ n (%)	24 (55.8)

^a^ Controller Therapy: treated with ICS or montelukast for more than 3 months

**Table 2:** Comparison of risk factors according to asthma outcome in wheezing children

Variables	Study group (43)		
	Non-asthma	Asthma	*p* value
Total n(%)	17(39.5)	26(60.5)	
Sex n(%)			
Male	10(58.8)	19(73.1)	0.329^*^
Female	7 (41.2)	7(26.9)	
Age group n(%)			
≤3 years old	10(58.8)	11(42.3)	0.289^*^
3-5 years old	7(41.2)	15(57.7)	
Systemic glucocorticoid n(%)			
Yes	6(35.3)	4(15.4)	0.254^*^
No	11(64.7)	22(84.6)	
Wheezing frequency n(%)			
<4 times	13(76.5)	8(30.8)	0.003^*^
≥4 times	4(23.5)	18(69.2)	
Age when first wheezing n(%)			
≤3 years old	17(100.0)	21(80.8)	0.139^*^
3-5years old	0(0.0)	5(19.2)	
Duration of wheezing onset to enroll, median (IQR)(years)	0.9(0.0,1.5)	2.1(0.4, 4.8)	0.052^**^
Allergic rhinitis n(%)	7(41.2)	15(57.7)	0.289^*^
Atopic dermatitis n(%)	9(52.9)	17(65.4)	0.415^*^
Family allergic diseases	6(35.3)	17(65.4)	0.053^*^
Asthma prediction index n(%)	9(52.9)	23(88.5)	0.024^***^
Inhalant allergen sIgE n(%)	8(47.1)	16(69.6)	0.151^*^
Food allergen sIgE n(%)	8(47.1)	15(65.2)	0.251^*^
Total IgE(IU/ml)	46(8.6, 204.0)	192.5(60.9, 805.0)	0.055^**^
CD4^+^CCR6^+^CRTh2^+^cells mean±SD(%)	0.8±0.6	1.6±0.8	<0.001^**^

*: Pearson Chi-Square; **: Mann-Whitney Rank sum test; **: t-test; ***: Continuity Correction

### Similar personal atopic features and family allergic diseases history between children with or without asthma diagnosis, but higher positive rate of loose API in children with asthma diagnosis

The percentage of AD and AR in asthma diagnosis group and without asthma diagnosis group were 65.4 %, 52.9 and 57.7 %, 41.2 % respectively (*p* > 0.05). There was no significant difference between those two groups in percentage of AR or AD. There was also no significant difference in family history of allergic diseases between the asthma (65.4 %) and the non-asthma (35.3 %) groups (*p* > 0.05) The positive rates of inhaled-allergen-specific IgE and food allergen-specific IgE in asthma group were 69.6 and 65.2 %, respectively, while the positive rates in non-asthma group were 47.1 and 47.1 %, respectively. The serum level of total IgE in wheezing children with or without asthma diagnosis were 46 (8.6, 204.0) IU/ml and 192.5 (60.9, 805.0) IU/ml, respectively. There was no significant difference in the presence of inhaled allergen-specific IgE (*p* > 0.05), food allergen-specific IgE (*p* > 0.05) and total IgE (*p* > 0.05) between the two groups. The positive rate of loose API in asthma diagnosis group and without asthma diagnosis group were 88.5 and 52.9 %, respectively. The positive rate of loose API was higher in asthma diagnosis group than without asthma diagnosis group (*p* < 0.05) (Table 2).

The duration of wheezing onset to enroll in asthma diagnosis group and without asthma diagnosis group were 0.9(0.0,1.5) years and years 2.1(0.4, 4.8) years, respectively. There was no significant difference between those two groups (*p* > 0.05) (Table 2).

### Similar positive rate of AD or AR history in children with or without recurrent wheezing

Thirty-six cases (83.7 %) had more than two wheezing episodes (recurrent wheezing) at the end of follow-up. 28 of 36 (77.8 %) cases with recurrent wheezing had either AD or AR history, whereas 5 of 7 (71.4 %) cases without recurrent wheezing had either AD or AR history. There was no significant difference between those two groups (*p* > 0.05, Pearson Chi-Square test).

### Higher circulation CD4^+^CCR6^+^CRTH2^+^ memory Th2 cells were associated with asthma diagnosis but not with AD or AR history

**Fig. 1:**
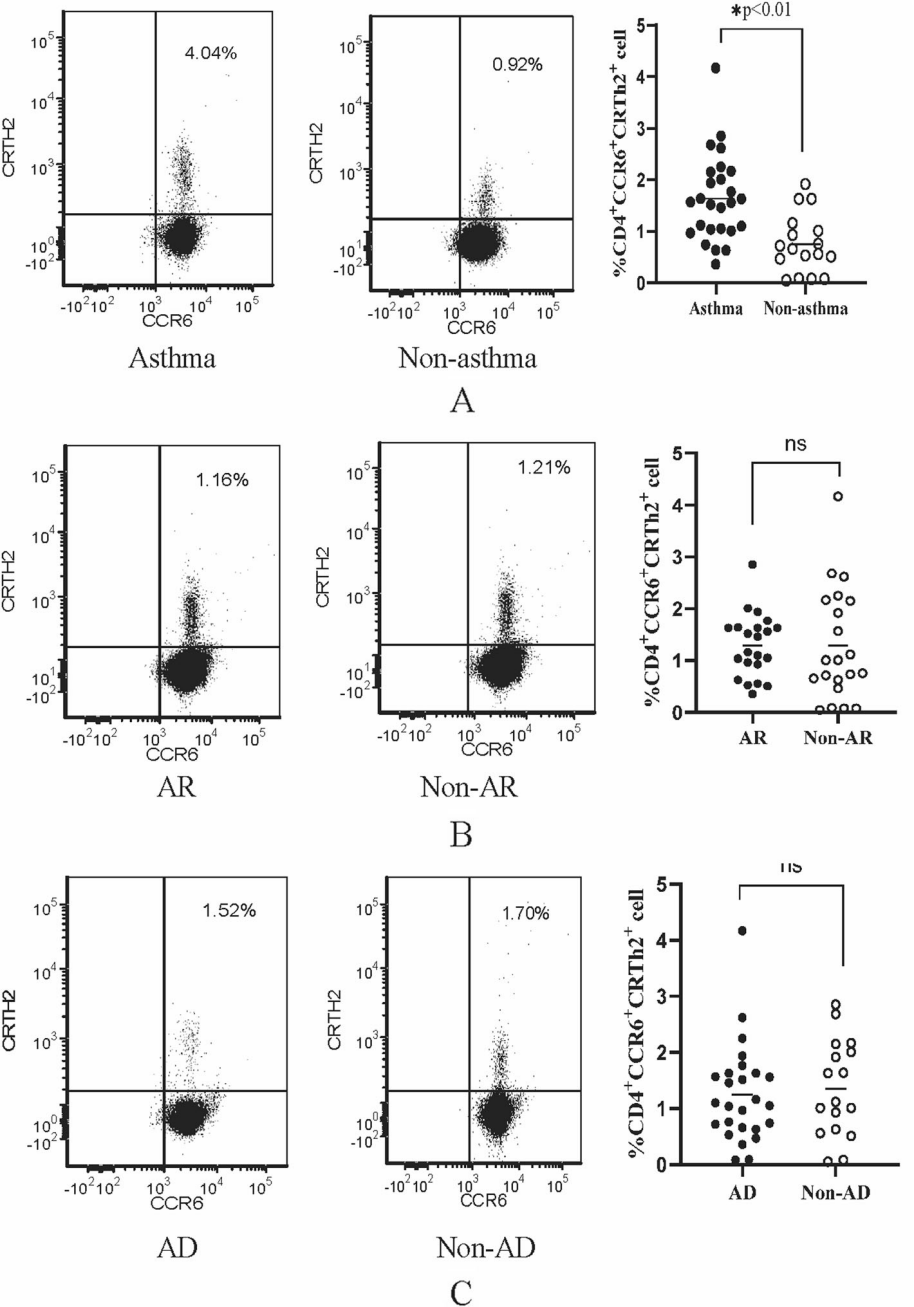
Flow cytometry anlysis of circulation CD4^+^CCR6^+^CRTh2^+^ memory Th2 cells. CD4^+^CCR6^+^CRTH2^+^memory Th2 cells were analyzed in CD4^+^ T cells. **a **Percentage of CD4^+^CCR6^+^CRTH2^+^memory Th2 cells in asthma diagnosed and without asthma diagnosed children. **b **Percentage of CD4^+^CCR6^+^CRTH2^+^ memory Th2 cells in AR history children. **c **Percentage of CD4^+^CCR6^+^CRTH2^+^memory Th2 cells in AD history children. All data expressed as mean±SD, T test was used to compare the difference between groups (*, *p* < 0.001)

CD4^+^CCR6^+^CRTH2^+^ cell is regarded as one type of pathologic memory Th2 cell which mainly resident in lung tissue. To investigate the difference of this type of memory Th2 cells between wheezing children diagnosed with or without asthma, peripheral blood CD4^+^CCR6^+^CRTH2^+^ memory Th2 cells were measured by flow cytometer at time of inclusion. Data were analyzed in CD4^+^ subset using CytExpert software. The results were used to correlate with diagnosis after the 2-year follow-up. In wheezing children diagnosed with asthma, the level of peripheral blood CD4^+^CCR6^+^CRTH2^+^ was 1.6 %±0.8 %, while the level in children without asthma was 0.8 %±0.6 %.There was a significant difference in the level of CD4^+^CCR6^+^CRTH2^+^ memory Th2 cells children with asthma compared with the non-asthma group (*p* < 0.01) (Table 2; Fig. 1).

We further explored the relationship between the level of CD4^+^CCR6^+^CRTh2^+^ memory Th2 cells and history of allergic diseases such as AR and AD. There was no significant difference in the level of CD4^+^CCR6^+^CRTh2^+^ cells between the AR group and the non-AR group(1.3 %±0.6 and 1.2 %±0.8 %) (*p* > 0.05), as well as between the AD group and the non-AD group(1.2 %±0.8 and 1.3 %±0.8 %) (*P* > 0.05, Fig. 1).

### The level of CD4^+^CCR6^+^CRTh2^+^ memory Th2 cells and wheezing frequency were effective indexes for the diagnosis of asthma in wheezing children

**Fig. 2:**
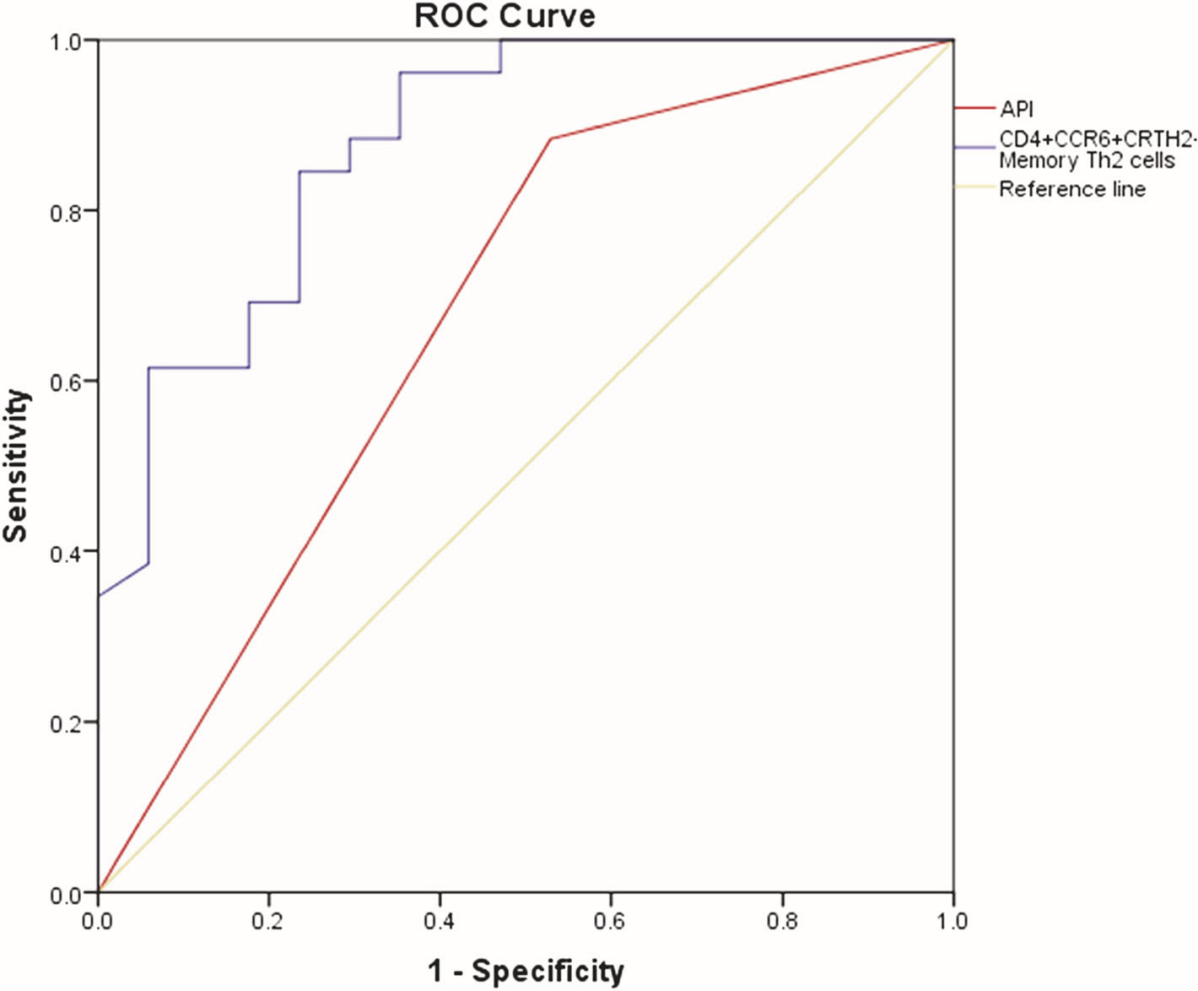
ROC curves of features for predicting asthma diagnosis. ROC curves of features for predicting asthma diagnosis using binary logistic regression analysis and the Wald X^2^ test. The area under the ROC curve for CD4^+^CCR6^+^CRTh2^+^memory Th2 cells and API were 0.879 (95%CI 0.774-0.984; *p*<0.001) and 0.678 (95%CI 0.505-0.850, *p*≤0.05)

Binary logistic regression analysis and the Wald X^2^ test were performed to identify the effective indexes for the diagnosis of asthma. We first analyzed several factors could affect the outcome of asthma diagnosis during follow-up using Pearson Chi-Square test and t-test (listed in Table 2). We found that there were significant differences in wheezing frequency,level of CD4^+^CCR6^+^CRTh2^+^ memory Th2 cells and API between children diagnosed with asthma and children with non-asthma outcomes. Binary logistic regression analysis and the Wald X^2^ test of each regression coefficient further showed that the level of CD4^+^CCR6^+^CRTh2^+^memory Th2 cells (EXP, 8.986; 95 % CI, 1.886–42.816; *P* < 0.01) and wheezing frequency (EXP, 0.127; 95 % CI, 0.023–0.703; *P* < 0.05) were risk factors for asthma in these children (Table 3). The ROC was adopted to calculate the sensitivity and specificity of the model. The area under the ROC curve for CD4^+^CCR6^+^CRTh2^+^ memory Th2 cells and API were 0.879 (95 %CI 0.774–0.984; *p* < 0.01) and 0.678(95 %CI 0.505–0.850,*p* ≤ 0.05) (Fig. 2). The cut-off value for CD4^+^CCR6^+^CRTh2^+^ memory Th2 cells is 0.575 %.

**Table 3:** Association between asthma and other variables using binary logistic regression analysis

Variable		B	S.E.	Wald	Sig.	Exp(B)	95%C.I. for Exp(B)	
							Lower	Upper
Step 1	Level of CD4^+^CCR6^+^CRTh2^+^ cells	2.040	0.683	8.928	0.003	7.688	2.017	29.299
	Constant	-1.880	0.791	5.651	0.017	0.153		
Step 2	wheezing frequency	-2.061	0.872	5.588	0.018	0.127	0.023	0.703
	Level of CD4^+^ CCR6^+^CRTh2^+^ cells	2.196	0.797	7.598	0.006	8.986	1.886	42.816
	Constant	-0.894	0.922	0.940	0.332	0.409		

### The level of CD4^+^CCR6^+^CRTh2^+^ memory Th2 cells displayed higher specificity and PPV in predicting asthma diagnosis

We calculated NPV and PPV for API and CD4^+^CCR6^+^CRTH2^+^ memory Th2 cells level in predicting asthma diagnosis. We regard the cases whose circulation CD4^+^CCR6^+^CRTh2^+^ cells level was higher than cut-off value as positive cases. The sensitivity, specificity, PPV and NPV of API in predicting asthma outcome were 88.5 %, 47.1 %, 71.9 and 72.7 % respectively. The same parameters of CD4^+^CCR6^+^CRTh2^+^ cells were 84.6 %, 76.5 %, 84.6 and 76.5 % respectively. The results indicated that circulation CD4^+^CCR6^+^CRTh2^+^ memory Th2 cells displayed higher specificity and PPV in predicting asthma diagnosis (Table 4).

**Table 4:** Performance of the API and circulation CD4^+^CCR6^+^CRTH2^+^ memory Th2 cells in predicting the outcome of children with wheezing episodes

Predictors	Sensitivity	Specificity	PPV	NPV
**Loose API**	88.5	47.1	71.9	72.7
**CD4**^**+**^**CCR6**^**+**^**CRTH2**^**+**^**cells**	84.6	76.5	84.6	76.5

*API* Asthma Predictive Index, *PPV* Positive predictive value, *NPV* Negative predictive value;

## Discussion

At present, the diagnosis and treatment of asthma under 6 years of age is mainly based on the risk of asthma. mAPI is a practical tool to evaluate the risk of asthma in clinic [6–8]. However, the positive predictive rate of mAPI usually around 60–70 % [9–11] which means 30–40 % of wheezing children with atopic feature will not develop asthma although atopy is a high-risk factor for asthma. In current study, we evaluated potential risk factor for asthma diagnosis.

First, we evaluated atopic features such as serum sIgE, total IgE, patient allergic disease history and family allergic disease between asthma diagnosed and without asthma diagnosed wheezing children since allergic diseases and family allergic diseases were used as an important indicator in evaluating the risk of asthma. In this study, 76.7 % of patients had history of AR or AD, 74.4 % of patients were positive for inhalants sIgE or food sIgE, which meant most of those patients had atopic feature. We found that none of those factors were significant difference between asthma diagnosed and without asthma diagnosed groups. Those results indicated that the value of above single factors in prediction of asthma in highly atopic children may limited. On the contrary, we compared wheezing profiles between asthma diagnosed and without asthma diagnosed wheezing children. We found that those diagnosed with asthma shown higher wheezing frequency during follow-up. Thus, wheezing frequency was more valuable in predicting of asthma diagnosis in atopic children. API is a practical tool for management younger wheezers in clinics. In this study, we evaluated loose API in predicting future asthma risk since part of participants had less than four wheezing episodes. The sensitivity and specificity of loose API were 88.5 and 47.1 %, respectively, indicated that API showed insufficient specificity in predicting asthma risk.

Next, we compared the difference of circulation CD4^+^CCR6^+^CRTh2^+^ memory Th2 cells between those two groups. We found that the level of CD4^+^CCR6^+^CRTh2^+^ memory Th2 cells in the asthma diagnosed group was significantly higher than those in the non-asthma diagnosed group. Logistic regression analysis further indicated that CD4^+^CCR6^+^CRTh2^+^ memory Th2 cells and wheezing frequency were the independent risk factor for the outcome of asthma diagnosis. As compared to loose API, circulation CD4^+^CCR6^+^CRTh2^+^ memory Th2 cells had better efficiency in predicting risk of asthma diagnosis based on higher specificity, higher PPV and larger area under the ROC curve.

Interestingly, there was no significant difference in the proportion of circulation CD4^+^CCR6^+^CRTh2^+^ memory Th2 cells in children with AR or AD history. These results suggest that the increase in circulation CD4^+^CCR6^+^CRTh2^+^ memory Th2 cells seemed specifically related to asthma but not AR or AD. Basic research indicated that CD4^+^CCR6^+^CRTh2^+^ memory Th2 cell is a type of pathogenic memory-type Th2 cell. Pathogenic memory-type Th2 cells are mainly distributed in local tissues for long periods of time and play key roles in the maintenance of chronic allergic inflammation [5, 12, 13]. CD4^+^CRTH2^+^CCR6^+^ cells were mainly found in the airway. CD4^+^CRTH2^+^CCR6^+^ cells secrete IL-17, IL-4 and IL-13, leading to the infiltration neutrophils and eosinophils, which is responsible for chronic inflammation associated with asthma. A small number of pathogenic memory-type Th2 cells could enter the circulation [5, 12]. Therefore, increasement of circulating CD4^+^CRTH2^+^CCR6^+^ memory Th2 cells may reflect the chronic airway allergic inflammation, an important immunological feature of asthma.

In summary, despite of small sample size, our exploratory study shown that higher wheezing frequency, but not history of allergic diseases or family allergic diseases were related to asthma diagnosis after 2 years follow-up in wheezing children who had atopic features. Moreover, circulation CD4^+^CCR6^+^CRTh2^+^ memory Th2 cells, which have certain lung tissue specificity, was increased in asthma diagnosed children and was risk factor for the outcome of asthma diagnosis. Detecting of CD4^+^CCR6^+^CRTh2^+^ memory Th2 cells in BALF would be more valuable in predict future asthma since those cells are mainly resident in airway. However current study had its limitation in sample size and short follow-up duration, so expansion of the sample size and prolonging the follow-up time is required to further validate the current results.

## Conclusions

Our exploratory study shown that circulating CD4^+^CCR6^+^CRTh2^+^ memory Th2 cells increased in asthma diagnosed children and it was a high-risk factor for asthma. Detection of this type of cells could be helpful in predicting the risk of asthma in wheezing children.

## Methods

### Study design and subjects

We recruited forty-three children under five years old who presented with recurrent wheezing or lower respiratory tract infection in respiratory ward or in clinic of Shanghai Xinhua hospital and Shanghai children’s Medical center affiliated to Shanghai Jiao Tong University School of Medicine from July 2017 to March 2018. All recruited children presented with wheezing or had history of at least one wheezing episode. Children had more than two wheezing episodes documented in medical record at the end of follow-up were regarded as recurrent wheeze. Asthma diagnosis was evaluated after the end of two-year follow-up. Wheezing was confirmed by doctors at the time of enrollment or from documentation in medical records. Exclusion criteria were: (1) bronchopulmonary dysplasia, tracheobronchial foreign bodies or congenital heart disease; (2) acute and chronic infectious diseases; (3) immunomodulatory treatment before or during the study; and (4) Children’s guardian has poor understanding of our study or had poor compliance after the preliminary assessment. Additionally, respiratory symptoms, using of systemic glucocorticoid or inhaled glucocorticoid within 7 day were recorded. The history of allergic diseases (allergic rhinitis (AR) and atopic dermatitis (AD) diagnosed by specialists according to their medical record and reviewed at the end of follow-up.) and family allergic history were collected through questionnaires filled out by the parents. At inclusion, peripheral blood was taken for allergen-specific IgE testing and determine the proportion of circulating CD4^+^CCR6^+^CRTh2^+^memory Th2 cells. The study was examined and approved by the Ethics Committee of Shanghai Xinhua hospital Affiliated to Shanghai Jiao Tong University School of Medicine, and the guardians of all included children provided signed informed consent.

### Laboratory tests

#### Measurement of serum allergen-IgE (sIgE) and total IgE

Serum allergen-specific IgE (aero-allergens and food allergens) and total IgE were detected using a DX-Blot 45 Automatic Western Blotting instrument (Hangzhou Zheda Dixun Biological Gene engineering Company, Zhejiang, China) according to the manufacturer’s instructions.

#### Detection of CD4^+^CCR6^+^CRTH2^+^ memory Th2 cells

Blood (100 µl), with heparin as anticoagulant, was mixed with PE-labeled anti-CRTH2(Biolegend, USA), PE-Cy5.5-labeled anti-CD4(Biolegend, USA) and PE-Cy7-labeled anti-CCR6(Biolegend, USA) monoclonal antibodies and incubated at room temperature avoiding light for 30 min. Red blood cells were removed by lysis, and CD4^+^CCR6^+^CRTH2^+^ memory Th2 cells were detected by CytoFLEX flow cytometry. Data analysis was performed using *CytExpert* version 2.3.0.84.

#### Follow up

The included children were followed up for 2 years in clinic. Patients required controller therapy visited clinic monthly. Otherwise, patients visited clinic when they had respiratory symptoms. All patients visited clinic at least once a year and received an annual telephone interview with their parents through questionnaires covered the frequency of wheezing episodes, pediatric respiratory specialists diagnosed asthma and medication for recurrent wheezing. Asthma diagnosis was made by pediatric respiratory specialists according to guidelines for the diagnosis and management of asthma in children (2016) which developed by the Chinese Pediatric Society, Chinese Medical Association [14] and Global Strategy for Asthma Management and Prevention(2020)(Chap. 6, diagnosis and management of asthma in children 5 years and younger). For children older than five years old, asthma diagnosis was based on typical respiratory symptom, lung function test and FeNO. For children less than five years old, asthma diagnosis was evaluated at the end of 2-year-follow-up by recurrent wheezing, response to bronchodilator, history of allergic disease, allergen sensitization, asthma in first-degree relatives and clinical improvement during 3 months of controller treatment (ICS or montelukast).

Since part of participants had less than four wheezing episodes, loose API was used to predict future risk of asthma. Loose API (asthma predictive index) were assessed according to detailed questionnaires, medical record information, serum sIgE, blood eosinophilic cell count. Participants with one major or two minor risk factors were defined as API-positive, otherwise were defined as API-negative. Briefly, physician-diagnosed atopic dermatitis, parental asthma, and sensitization to inhalant allergen were defined as major risk factors. Minor risk factors included sensitization to food allergens, eosinophil levels in the peripheral blood eosinophilic cell ≥ 4 %, and wheezing unrelated to a cold.

### Statistical analysis

All statistical analysis was carried out using *SPSS v. 22.0*. Continuous variables were represented by mean ± SD or median (interquartile range, IQR). Classification variables were represented by frequency. T test was used to compare the normally distributed data. Mann-Whitney rank sum test was used to compare the non-normally distributed data. Chi-Square test was used to compare the difference of qualitative variables between children diagnosed with or without asthma. Binary logistic regression analysis and the Wald X^2^ test was used for correlation analysis. The area under the curve was calculated by the ROC of the subjects. Bilateral significance level was set at *P* < 0.05.

## Data Availability

All data generated or analyzed during this study are stated in this published article.

## Abbreviations

AD: Atopic dermatitis
AR: Allergic rhinitis
sIgE: Specific IgE
API: Asthma predictive index
